# Brief review: Applications of nanocomposite in electrochemical sensor and drugs delivery

**DOI:** 10.3389/fchem.2023.1152217

**Published:** 2023-03-16

**Authors:** Zia Ul Haq Khan, Taj Malook Khan, Amjad Khan, Noor Samad Shah, Nawshad Muhammad, Kamran Tahir, Jibran Iqbal, Abdur Rahim, Syed Khasim, Iftikhar Ahmad, Khadija Shabbir, Noor Shad Gul, Jianbo Wu

**Affiliations:** ^1^ Department of Environmental Sciences, COMSATS University Islamabad, Vehari, Pakistan; ^2^ Drug Discovery Research Center, Southwest Medical University, Luzhou, China; ^3^ Department of Pharmacology, Laboratory of Cardiovascular Pharmacology, The School of Pharmacy, Southwest Medical University, Luzhou, China; ^4^ Department of Zoology, University of Lakki Marwat, Lakki Marwat, Pakistan; ^5^ Department of Dental Materials, Institute of Basic Medical Sciences, Khyber Medical University, Peshawar, Pakistan; ^6^ Institute of Chemical Sciences, Gomal University, Dera Ismail Khan, Pakistan; ^7^ College of Natural and Health Sciences, Zayed University, Abu Dhabi, United Arab Emirates; ^8^ Department of Chemistry, COMSATS University Islamabad, Islamabad, Pakistan; ^9^ Nanotechnology Research Unit, Faculty of Science, University of Tabuk, Tabuk, Saudi Arabia; ^10^ Department of Physics, Faculty of Science, University of Tabuk, Tabuk, Saudi Arabia

**Keywords:** nanotechnology, nano-formulations, nano-systems, diagnosis, targeted drug delivery, treatment of disease

## Abstract

The recent advancement of nanoparticles (NPs) holds significant potential for treating various ailments. NPs are employed as drug carriers for diseases like cancer because of their small size and increased stability. In addition, they have several desirable properties that make them ideal for treating bone cancer, including high stability, specificity, higher sensitivity, and efficacy. Furthermore, they might be taken into account to permit the precise drug release from the matrix. Drug delivery systems for cancer treatment have progressed to include nanocomposites, metallic NPs, dendrimers, and liposomes. Materials’ mechanical strength, hardness, electrical and thermal conductivity, and electrochemical sensors are significantly improved using nanoparticles (NPs). New sensing devices, drug delivery systems, electrochemical sensors, and biosensors can all benefit considerably from the NPs’ exceptional physical and chemical capabilities. Nanotechnology is discussed in this article from a variety of angles, including its recent applications in the medical sciences for the effective treatment of bone cancers and its potential as a promising option for treating other complex health anomalies *via* the use of anti-tumour therapy, radiotherapy, the delivery of proteins, antibiotics, and vaccines, and other methods. This also brings to light the role that model simulations can play in diagnosing and treating bone cancer, an area where Nanomedicine has recently been formulated. There has been a recent uptick in using nanotechnology to treat conditions affecting the skeleton. Consequently, it will pave the door for more effective utilization of cutting-edge technology, including electrochemical sensors and biosensors, and improved therapeutic outcomes.

## Introduction

Nanotechnology is used in many areas, including surface engineering, biotechnology, electrical engineering, and regenerative medicine. Sometimes common macro-materials can be synthesized into Nano-sized particles with entirely new sets of physical and chemical characteristics. If, for example, the particle size is 100 nm or less, the quantum size effect becomes more pronounced (Tahir et al., 2016b). When particles of a material are shrunk down to the nanoscale, they take on new electrical properties as a result of the principle, and they may now contain conductive elements that were previously insulators in their macroscale form. In addition to potential shifts in electrical properties, an increase in surface area to volume ratio may cause modifications in mechanical characteristics. Because of their increased surface area to volume ratio, anaphase materials interact with nearby structures more strongly. Nano biomaterials, because of their physiochemical properties, might be used in tumour diagnosis and treatments like tissue regeneration or therapy (Han et al., 2015; Han et al., 2016). Nano biomaterials and extensive research into their functionality should be used to forge a bridge between tumour therapy and regenerative medicine.

Surgical excision, where the tumour is removed, is the mainstay of cancer treatment, followed by chemotherapy to eliminate any chance of tumour recurrence (Ali et al., 2017; Wang X. et al., 2017). One major issue for diabetic patients is that the surgery can lead to problems with tissue regeneration. Fibrous electrospun scaffolds have been shown to mimic the extracellular matrix (ECM) and promote cell growth and differentiation in regenerating tissues like bone, skin, nerve, and heart (Khan et al., 2016b). Surface modifications made with Nano biomaterials on implants also aid this process (Karazisis et al., 2016). Specifically, in orthopedics, this adjustment allows for interaction with the bone, leading to more efficient Osseo integration (Mattei and Rehman, 2015). Nanoparticles can effectively target sites at the subcellular level because of their Nano-size (Ahmad et al., 2015). In orthopedics, hydroxyapatite and collagen make up the nanoscale structure of bone. (Khasim et al., 2021). All sorts of medical goods have been made more accessible and practical thanks to the implementation of these projects and the recognition of their interconnectedness. For effective drug management and the establishment of *in vitro* diagnostic (Shi et al., 2010; Tahir et al., 2015b; Sherpa et al., 2020), nanotechnology is being applied in a “Nano medicine” fashion. Nanotechnology’s applications in healthcare are growing at a dizzying rate, particularly in the fields of diagnosis and drug delivery. (Verma and Rotello, 2005; Han et al., 2007; Ghosh et al., 2008; Bajaj et al., 2010; Al-Hartomy et al., 2019; Liu et al., 2019a; Sherpa et al., 2020).

**FIGURE 1:**
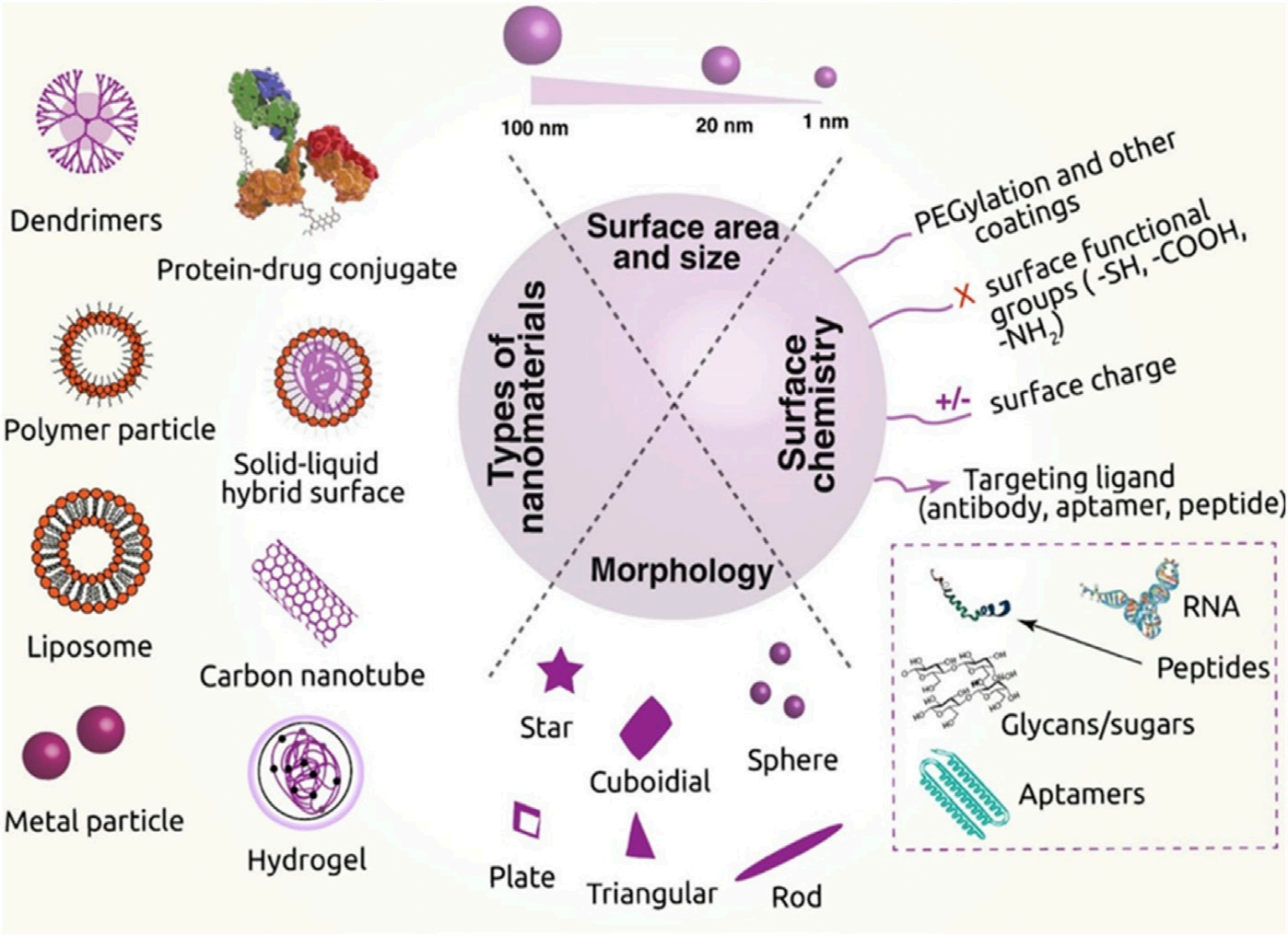
Various nanomaterials used in cancer therapy, their critical physical features, and the surface chemistry needed to transport drugs are depicted in this diagram. [Reprinted with permission from Nanomaterial MDPI, at2020].

Nanotechnology has been used in recent years better to deliver drugs to their intended sites of action (Tahir et al., 2015b; Kumar et al., 2020; Peer et al., 2020; Zaman et al., 2022). Nanoparticles can be constructed from a wide range of materials, including organic compounds, inorganic compounds, lipids, and proteins, all of which are effective at targeting tumours (Figure 1) (Kumar et al., 2020). Nanocarriers are useful in therapeutics for a number of reasons, such as i) their inertness to common drug-delivery challenges like solubility and stability and ii) their ability to protect drugs from degradation by enzymes like protease, thereby extending their half-lives inside the body. Several medications can be delivered at once, iii) the transport and delivery of medicines are greatly improved, iv) drug release at the targeted cancer sites is guaranteed, and v). The transportation and delivery of multiple medications are facilitated. As a result, nanotechnology promotes the development of novel materials that enhance the delivery of drugs, thus revolutionizing cancer pharmacology (Kim et al., 2012; Sun et al., 2017; Kumar et al., 2020; Zaman et al., 2021).

### Inorganic nanoparticles

Modern drug delivery systems rely heavily on this class of nanoparticles because of their precisely controlled physical properties, including size and shape tuning, tunable physicochemical properties, controlled surface chemistry, and a wide range of multi-functionalities. As of late, inorganic nanoparticles with particular properties have been manufactured and used in various biological settings, most notably in the treatment and management of cancer. Due to their distinctive properties and recent advances in fundamental understanding (Khan et al., 2016b). Metal nanoparticles and metal oxides have risen to prominence among inorganic nanoparticles. Effective drug delivery using carbon-based nanostructures and mesoporous silica nanoparticles has recently received much attention. Various inorganic Nanocarriers for anticancer drug delivery are listed in Table 1. Drugs carried by inorganic nanomaterials are listed in the table, along with details about the type of nanomaterial used and the targeted tumor cells.

**TABLE 1:** Illustrates the type of Nanocarriers, the drug used, and the target site.

Nanocarriers	Materials	Drug	Target	Refs
Metal NPs	Pluronic-b-poly (l-lysine) and gold nanoparticles	Paclitaxel	Human breast cancer (*in vitro*/*in vivo*)	Liu et al. (2019a)
	Folic acid, transferrin, and gold nanoparticles	Gemcitabine	Human mammary gland breast adenocarcinoma (*in vitro*)	Al-Hartomy et al. (2019)
	Apatite stacked gold nanoparticles	Docetaxel	Human liver cancer (*in vitro*)	Safwat et al. (2018)
Carbon NPs	PEG and single-walled carbon nanotubes	Cisplatin	Head and neck cancer (*in vitro*/*in vivo*)	Nagesh et al. (2016)
	PEG, anionic polymer, dimethyl maleic acid, and carbon dots	Cisplatin IV	Human ovarian carcinoma (*in vitro*/*in vivo*)	Nagesh et al. (2016)
	Endoglin, iron, single-walled carbon nanotubes	Doxorubicin	Murine breast cancer (*in vitro*/*in vivo*)	Bhirde et al. (2010)
	Carbon nanoparticles	Methotrexate	Human lung carcinoma (*in vitro*)	Feng et al. (2016)
	Human serum albumin, single-walled carbon Nanotubes	Paclitaxel	Human breast cancer (*in vitro*)	Qin et al. (2015)
	Carboxymethyl chitosan, fluorescein isothiocyanate, lactobionic acid, and graphene oxide	Doxorubicin	Human hepatocarcinoma (*in vitro*)	Al Faraj et al. (2015)
	Dendrimer, gadolinium diethylene triamine pentaacetate, prostate stem cell antigen monoclonal antibody	Doxorubicin	Prostate cancer (*in vivo*)	Ajmal et al. (2015)
Mesoporous silica NPs	PEG, amino-β-cyclodextrin, folic acid, mesoporous silica NPs	Doxorubicin	Breast cancer (*in vivo*)	Shao et al. (2015), Gao et al. (2019)
	Lanthanide doped upconverting nanoparticle, mesoporous silica nanoparticles	Doxorubicin	Murine hepatocellular carcinoma (*in vitro*/*in vivo*)	Peng et al. (2012), Gao et al. (2019)
	Bismuth III) sulphide nanoparticles, mesoporous silica nanoparticles	Doxorubicin	Multidrug-resistant breast cancer (*in vitro*/*in vivo*)	Wen et al. (2012), Pan et al. (2016)
	(S)-2-(4 isothiocyanatobenzyl)-1,4,7-triazacyclononane-1,4,7-triaceticacid, PEG, Hollow mesoporous silica nanoparticles	Sunitinib	Human glioblastoma (*in vitro*/*in vivo*)	Zhang et al. (2014)
	Poly (2-(diethylamino)ethyl methacrylate), Hollow mesoporous silica nanoparticles	Doxorubicin	Human cervical epithelial malignant carcinoma (*in vitro*)	Liu et al. (2013)

Recent advances in bio-nanotechnology have allowed for the emergence of several novel approaches to treating bone tumors. The dilemma of treating bone tumors lies in achieving cancer control and bone regeneration. Many years of careful interdisciplinary study (Liu et al., 2019b; Xue et al., 2019) are needed to develop methods that can solve these problems and ultimately cure the ailment. Researchers have put in a lot of time and effort to come up with a new approach to these problems, one that may aid in bone regeneration and reduce the likelihood of tumor recurrence. There are some promising developments in the search for a cure for bone tumors, but the research is still in the early stages (Xue et al., 2019).

## Nanotechnology in cancer diagnosis

Cancer has surpassed all other diseases as the primary public health crisis around the world. In 2018, researchers anticipated that 18.1 million new cases of concern would be reported and 9.6 million deaths would be attributed to cancer (Xiong et al., 2015). In addition, it is a large group of diseases that arise when abnormal cells multiply uncontrollably and spread to other parts of the body, ultimately resulting in the patient’s death. Therefore, it is crucial to detect cancer early so its spread can be halted, and the disease can be treated to prevent mortality. As one of the most widely used methods, nanotechnology has become increasingly significant in cancer research. The benefits of this method can be seen in various cancer-related fields, including diagnosis, treatment, drug delivery, gene therapy, biomarker mapping, targeted therapy, molecular imaging, and more (Niemelä et al., 2015). Gold nanoparticles and quantum dots, both products of nanotechnology, play a crucial role in the molecular detection of cancer tumors at early stages in patients (Niemelä et al., 2015). Biomarkers are a type of nanotechnology-based molecular diagnostic that allows for a quick and accurate cancer diagnosis (Mondlane et al., 2021). Thanks to advancements in nanotechnology, drugs can now be delivered to a precise location within the body without harming any neighboring cells (Ahmad et al., 2016; Tran et al., 2017; Hu et al., 2018). For this reason, nanomaterials with a biological origin can cross cellular barriers easily (Ahmad et al., 2016; Chaturvedi et al., 2019).

**FIGURE 2:**
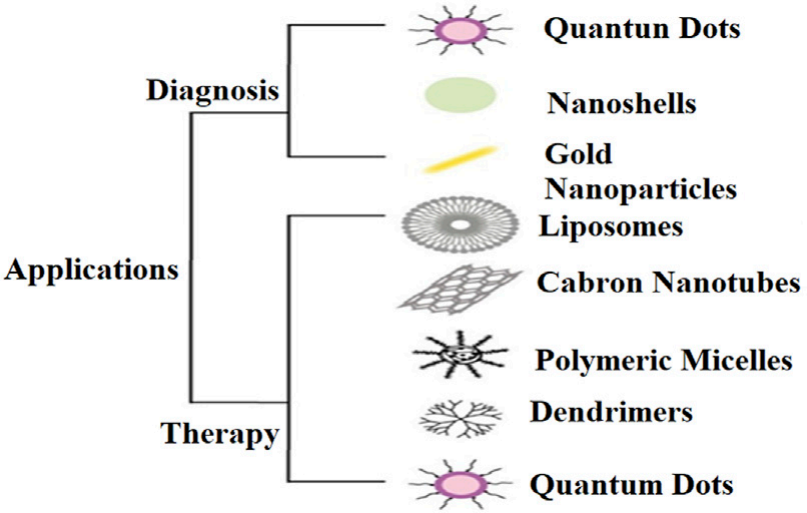
Nanoparticle usage for diagnosis and therapy of cancer.

For quite some time, nanomaterials’ active and passive targeting abilities have made them useful in cancer treatment. Many drugs can be used to treat cancer, but their effectiveness varies depending on the type of cancer being treated. Besides being ineffective, this approach has many unintended consequences, some of which can harm healthy cells. For this reason, many studies (Hu et al., 2018; Chaturvedi et al., 2019). Have concluded that combining various nanomaterials, such as polymers, liposomes, *etc.*, can improve the effectiveness of cancer treatment design while eliminating relative drug toxicity. Much more study is required before nanomaterials are widely accepted for use in the clinical treatment of cancer, however, because of their potential toxicity (Tahir et al., 2015c; Chaturvedi et al., 2019). This study will investigate the use of nanotechnology in cancer diagnosis and treatment, focusing on its advantages and disadvantages (Figure 2). Eventually, malignant tissues develop when specific changes in the DNA of genes cause abnormalities in the composition and functionality of some cells, leading to uncontrolled, abnormal cell growth. Cancer can be divided into two broad categories: benign and malignant. Benign tumors do not spread beyond their original site, while malignant tumors produce abnormal cells that can infect neighboring tissues and organs. Suppressing the growth and spread of abnormal cells, as well as detecting cancer at an early stage, are the primary goals of cancer treatment strategies (Bharali and Mousa, 2010; Tahir et al., 2015c). The main aims of cancer treatment strategies are identifying the disease in its initial stages and suppressing the production and spread of abnormal cells (Bharali and Mousa, 2010). For this purpose, various techniques such as computed tomography (CT), positron emission tomography (PET), magnetic resonance imaging (MRI), and ultrasound are highly utilized (Bharali and Mousa, 2010; Kim et al., 2010). However, clinical data on different cancer types and stages severely limits the utility of such imaging systems. As a result, it can be challenging to get a complete picture of the patient’s health condition on which to base the best treatment (Wang et al., 2005; Kim et al., 2010; Akhter et al., 2013; Rahim et al., 2014).

### Nanoparticles cytotoxicity to osteosarcoma cells

It has been suggested in several scientific papers that NPs can inhibit tumor growth. Rahim et al. discovered that in Saos-2 (osteosarcoma) cells, cell viability could be easily reduced by the use of 24.3 nm gold nanoparticles (AuNPs) capped with advanced glycation products; it also triggers apoptosis (Rahim et al., 2014; Khan et al., 2016a). In addition, the anticancer activity of AuNPs is shape-dependent, with rod- and star-shaped AuNPs being more effective against osteosarcoma cell lines 143b and MG63 than spherical AuNPs. (Khan et al., 2016a; Nayak et al., 2016). A wide variety of other nanoparticles also exhibit anticancer activity. There is evidence that Ag NPs can inhibit the proliferation of MG63 (osteosarcoma) cells [56]. Furthermore, it is unclear whether the nanoparticle size is responsible for the effects seen or whether silver’s presence is to blame. Interestingly, 15–34 nm Ag NPs threaten A-431 (osteosarcoma) cells more than AgNO_3_ [56]. (Nayak et al., 2016). In a similar vein, Kovacs et al. found that citrate-Ag NPs with diameters of 5 nm and 35 nm affected the viability of two osteosarcoma cell lines (U_2_OS and Saos-2). (Kovács et al., 2016; Barani et al., 2021). To his surprise, he found that the cytotoxicity of nanoparticles increased as their size decreased. Moreover, Ag NPs were thought to be more effective than cisplatin at the same concentration in inhibiting cell proliferation. Ag NPs initiate mitochondrial stress, which in turn triggers apoptosis. Unfortunately, no historical data explains how Cu NPs become cytotoxic to osteosarcoma cells. However, we could not find any data indicating that iron or aluminum nanoparticles affect osteosarcoma cells. There is evidence to suggest that metal oxide nanoparticles may have anticancer effects. TiO_2_NPs with a mean particle size of 3.8 nm were shown to be cytotoxic to U_2_OS cells at concentrations greater than and for more extended periods than 0.5 g/mL. Through suppressing ROS production and lowering glutathione (GSH) levels, TiO_2_NPs induced oxidative stress (Palza et al., 2017). The concerned toxicity of TiO_2_NPs was also committed in another study. Di Virgil (Baker et al., 2009) explored more of the anticancer functions of 15 nm TiO_2_NPs and 50 nm aluminum oxide nanoparticles (Al_2_O_3_NPs). Using the MTT assay, we found that both nanostructures were cytotoxic to UMR-106 cells at concentrations greater than 50 mg/mL (Baker et al., 2009; Jabir et al., 2012).

One factor that determines how vulnerable cancer cells are to nanoparticles is the surrounding environment’s pH. Results against osteosarcoma cells treated with dextran-coated cerium oxide nanoparticles (CeO_2_NPs) were better at acidic pH (pH = 6) than at neutral pH (pH = 7) or basic pH 9. Under the same conditions, the cytotoxicity of CeO_2_NP towards healthy bone cells was extremely low. One possible mechanism for the cytotoxicity of CeO_2_NPs is an increase in reactive oxygen species (ROS) production (Jabir et al., 2012; Tahir et al., 2015a). In addition, another study discovered that (ZnONPs) may be harmful to MG63 because they can stimulate ROS synthesis (Tahir et al., 2015a). Osteosarcoma could be treated with more than just metal nanoparticles. Many different therapies, not just metal nanoparticles, have shown promise in treating osteosarcoma. At 1–8 mg/mL concentrations, the nanoparticle (100 nm) Fucoid induced apoptosis in 143B cells, reducing their viability (Tahir et al., 2015a; Sisubalan et al., 2018). Better anticancer efficacy was seen with nanoparticles of Fucoid compared to macro-size Fucoid and in an *in vivo* osteosarcoma model in CH_3_ mice (Sisubalan et al., 2018). Similarly, to the *in vitro* model, Fucoidan nanoparticles induced apoptosis in osteosarcoma when applied *in vivo*. Furthermore, Fucoidan NPs should pose little to no health risks because they do not affect animal body weight (Kimura et al., 2013; Sisubalan et al., 2018). It was also discovered that hydroxyapatite nanoparticles (HA-NPs) have favorable outcomes.

In particular, HA-NPs are intriguing because of their similarity in composition and structure to bone (Qing et al., 2012; Kimura et al., 2013). It has been discovered that HA-NPs cause apoptosis in MG63 cells while increasing the survival of healthy osteoblasts (Qing et al., 2012; Kimura et al., 2013). Aside from causing selective cytotoxicity in cancer cells, HA-NPs also caused changes in the cells’ ultrastructure. Nuclei showed morphological changes, and mitochondria were found to be enlarged; ribosomes had become dissociated from RER, and mitochondria had enlarged. (Abrams et al., 2006; Qing et al., 2012; Kimura et al., 2013).

For a fuller picture of NPs’ biological effects, it is essential to learn whether or not they are internalized (Abrams et al., 2006; Di Virgilio et al., 2010). In conclusion, as shown in Table 2, increased ROS generation by different nanoparticles (metal, metal oxide, HA) can mediate anticancer activity. In addition, the size, shape, type, and capping activities of NPs can all affect their anticancer activity. Osteosarcoma tumor cells can take in and store a variety of nanoparticles. According to Azarami et al., the uptake of gelatin nanoparticles by 143B cells varies from 112 to 303 nm. When nanoparticles were more significant, they were absorbed less effectively (Abrams et al., 2006). MG63 cells were also demonstrated to be capable of internalizing 100 nm PGLA NPs Internalization of 100 nm PGLA NPs by MG63 cells was also demonstrated.

**TABLE 2:** Summary of nanoparticles (NPs) effects *in vitro* model of osteosarcoma.

Nanoparticles type	Osteosarcoma cell line	Effect	Additional comment	References
Gold NPs 24.3 nm capped with advanced glycation products	Saos-2	Cytotoxicity		Wang et al. (2005), Chen et al. (2019)
Gold NPs rods Gold NPs stars Gold NPs spheres	143B	Cytotoxicity Apoptosis induction	Cytotoxicity was shape-dependent	Khan et al. (2016a), Bhushan et al. (2017)
	MG63			
Citrate silver NPs5nm and 35 nm	U2OS	Cytotoxicity Proliferation Inhibition Mitochondrial stress and apoptosis induction	Cytotoxicity size-dependent	Khan et al. (2016a), Gu et al. (2013)
	Saos-2		NPs were more effective than cisplatin	
Copper NPs 10 nm	Saos-2	Cytotoxicity	_	Chen et al. (2018)
Titanium oxide NPs15 nm	UMR-106	Cytotoxicity NPs were present in phagocyte chemical	_	Zhang et al. (2017)
Aluminum oxide NPs50 nm	UMR-106	Within the cells, Cytotoxicity NPs were present in phagocyto chemical	Cytotoxicity wash-dependent	Venturoli and Rippe (2005), Zhang et al. (2017)
Dextran-coated cerium oxide NPs 3–4 nm	MG63	Within the cells, Cytotoxicity Increased ROS production	Cells were more susceptible to NPs in an acidic environment	Matsumura and Maeda (1986), Venturoli and Rippe (2005)
Zinc oxide NPs22 nm	MG63	Cytotoxicity Increased ROS production Apoptosis induction	_	Matsumura and Maeda (1986), Dreher et al. (2006)
Cerium oxide NPs26 nm	MG63	Cytotoxicity Increased ROS production Apoptosis induction	_	Nomura et al. (1998), Dreher et al. (2006)
Fucoidan NPs100 nm	C3H	Cytotoxicity Apoptosis induction	Fucoidan in NPs was more effective than Fucoidan itself	Nomura et al. (1998), Hu-Lieskovan et al. (2005)
Hydroxyapatite NPs40 nm	MG63	Selective cytotoxicity Only to cancer cells Ultrastructure changes	HA-NPs are cytotoxic to osteosarcoma and stimulate the growth of healthy osteoblast	Chen et al. (2005), Hu-Lieskovan et al. (2005)

### Single photon emission computed tomography (SPECT)/CT imaging SPECT

**FIGURE 3:**
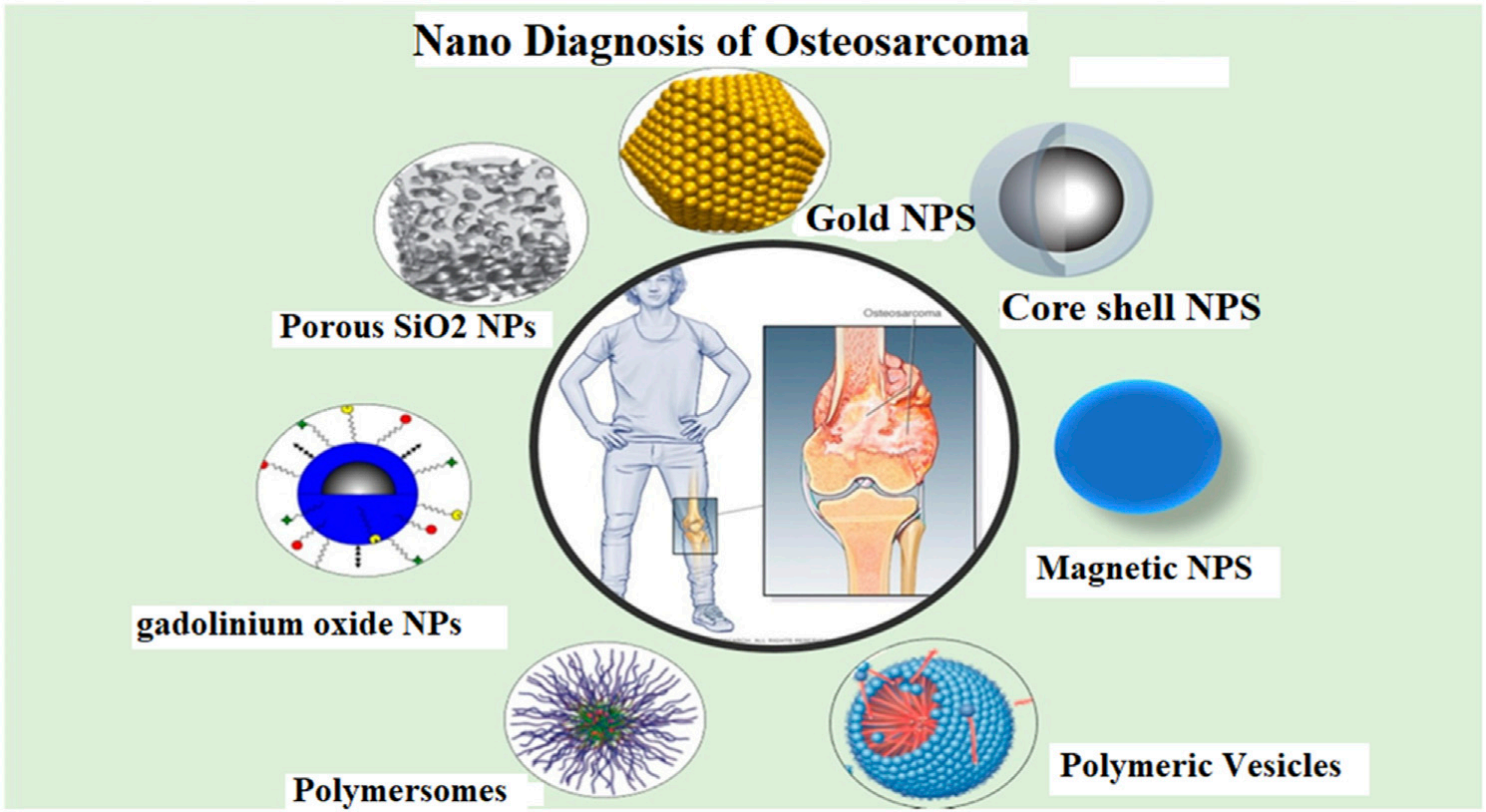
Different nanoparticles for diagnosis of osteosarcoma (OSA). [Reprinted with permission from Biosensors, MDPI, at2021].

For many years, it has served as a focal point for researchers in nuclear medicine. The use of SPECT and CT together has increased in popularity in recent years and is effective in various clinical settings. As shown in Figure 3 (Chen et al., 2005), the mixture has a high penetrating capability and is better suited to imaging deep tissue, especially OSA imaging. Diagnosing malignant osteocytes *via* multifunctional nanomaterials may also be helpful for treatment and diagnosis. It has been investigated whether or not albumin-based Nanomedicines could be beneficial in cancer treatment (Abrams et al., 2006; Poilil Surendran et al., 2018). Due to its imaging and therapeutic capabilities, nanomedicine may replace traditional therapeutic navigation and monitoring methods.

For targeted delivery and treatment monitoring, Chen et al. developed albumin-based gadolinium oxide nanoparticles, loaded them with doxorubicin, and coupled them with alendronate (Chen et al., 2018). Moreover, the authors noticed an excellent NPs distribution throughout the body after SPECT imaging and radio labeling with 125I. SPECT imaging revealed increased bone tumor formation and longer NPs retention in bone cancer (Chen et al., 2018). CT imaging and pathological investigation, on the other hand, revealed that the combination therapy used in this trial was beneficial (Nomura et al., 1998). According to the results of this investigation, albumin-based nanoparticles could be used to image and assess bone conditions. In recent years rapidly, metallic nanoparticles have emerged as a valuable tool in a variety of fields, including medicine and biosensing. Metallic NPs show little cytotoxicity in normal cells and are well-targeted and localized at the subcellular level or where cancer originates. Furthermore, the size and structure of nanoparticles help them exhibit their best performance in drug delivery to patients with critical issues like OSA. Platinum NPs are widely acknowledged as secure, stable, and productive due to their potent sensing abilities and plasmonic features (Chen et al., 2005).

### Critical properties of anticancer nanoparticles

As a rough estimate, nanostructures used to treat cancer are between 10 and 100 nm in size. Considering that the kidney has a 10 nm elimination threshold, the sieving coefficient of the glomerular capillary walls should be used as the lower bound (Chen et al., 2005) (Giljohann and Mirkin, 2009). Currently, the diameter of the upper limit is not specified. Moreover, macromolecule leakage in tumor vasculature is a well-documented phenomenon. Animal models of tumors have a dysfunctional lymph system, contributing to the ERP effect of accumulated macromolecule leakage from blood vessels (Giljohann and Mirkin, 2009; Cheng et al., 2012). Multiple pieces of evidence demonstrate the existence of this phenomenon in human beings. Therefore, it is not good to use particles with a diameter of less than 100 nm because they might escape from the blood vessels. However, phagocytes in the body may be able to destroy larger molecules (Cheng et al., 2012; Colson and Grinstaff, 2012). Experiments on animal models reveal that neutral or slightly negatively charged entities with a diameter of less than 150 nm can travel through tumor tissue (Colson and Grinstaff, 2012; Du et al., 2020).

Furthermore, recent findings demonstrate that nanoparticles with a minimal positive charge in the 50–100 nm size range can penetrate giant tumors after systemic injection (Du et al., 2020). When these nanoparticles of size ranging between 10 and 100 nm are added to the blood circulation flow, these being slightly charged, either positive or negative, reach the tumor site. At this diameter range, these nanostructures could not escape normal vasculature (which requires diameters less than 12 nm). Still, there is a possibility that they might travel to the liver because entities of such size (100–150 nm) are capable of it (Danhier et al., 2012; Du et al., 2020).

### Nanoparticle surface properties

Compared to larger molecules, nanostructures have a much higher surface-to-volume ratio, which can be harnessed for human benefit in various contexts (Danhier et al., 2012). The fate of a nanoparticle depends upon how it interacts with its surroundings, which further depends upon its size and shape Self–Self and Self–non–self-interactions are negligible in nanoparticles that are sterically stabilized (for example, by polyethylene glycol (PEG) polymers on their surface) and are in an ionic form which means they possess either slightly positive or negative (Danhier et al., 2012). ]. Even more so, many negatively charged components are inside the vessels and on the outer or inner surfaces of cells, which would repel NPs with a negative charge (Danhier et al., 2012; Papasani et al., 2012).

Scavenging by macrophages and subsequent clearance by the reticuloendothelial system increases in tandem with growing surface concentrations. Consequently, limiting non-specific interactions through steric stabilization and surface charge control aids in preventing nanoparticle loss to undesirable places (Papasani et al., 2012). While it is currently impossible to eliminate non-specific interactions, some particle loss will always occur even with this method; the goal here is to minimize the interactions as much as possible. If nanoparticle loss could be stopped, the nanoparticles inside a mammal would be spread out evenly if there were no size limits due to thermodynamics. Non-etheless, there may be inconsistency due to size limitations in several locations throughout the body. For instance, the blood-brain barrier prevents harmful substances from entering the brain by setting considerable size and surface properties standards. Understanding the size and surface quality requirements for reaching these sites allows for the localization of nanoparticles to specific locations within the body.

### Nanotechnology and targeted drug delivery for bone diseases

Nanotechnology’s permeation into every aspect of our lives, from the medical to the environment, is undeniable. As a result, nanomaterials are being explored for use in medical devices to improve treatment efficacy and patient safety]. They add to the tools available for studying diseases through diagnostic imagery and medicine (Elazar et al., 2010; Herma et al., 2019)]. Nanoparticles can also provide a fine tissue regeneration structure (scaffold), revolutionizing medical tissue technology (Herma et al., 2019). Human bone disease encompasses many skeletal anomalies, including malformations that significantly affect mobility and mortality. As of yet, there is no cure for many diseases and disorders affecting the skeleton, including arthritis, osteosarcoma, bone cancer, and others. There is an immediate need for novel pharmaceuticals and drug delivery systems for the safe and effective clinical treatment of rheumatoid arthritis, osteoarthritis, osteosarcoma, and metastatic bone cancer (Herma et al., 2019). Obtaining a treatment method for bone cancer that guarantees bone regeneration and does not pose any adverse effects while removing the tumor is challenging. Researchers have investigated a targeted application of nanotechnology to address these challenges and enhance the efficacy of therapy. Tumour incidence and mortality remain high over the world. A focused application of nanotechnology has been explored to discover a possible strategy to overcome these issues and improve therapy effectiveness.

Over 14 million individuals worldwide are diagnosed with cancer yearly, and eight million will inevitably succumb to it. Traditional methods of treating cancer, such as radiation therapy and chemotherapy, have been shown to have several undesirable side effects and to be less effective than other options. There is a lack of satisfaction with current tumor treatments, so scientists are looking for alternatives that can effectively target and kill tumor stem cells with few unintended consequences. In addition, novel approaches to tumor therapy, such as immunotherapy, targeted therapy, physical ablation, gene therapy, photodynamic therapy (PDT), and photothermal therapy (PTOT), have already demonstrated clinical efficacy. All these treatment approaches share a common characteristic: they necessitate the involvement of a carrier (Israel et al., 2020; Woodman et al., 2021). Since viral carriers can cause insertional mutagenesis and immunogenicity, creating a safe and effective carrier is crucial. Due to their biocompatibility, Nano size, medicinal substance fills up, and material characteristics, nanocomposites are quickly being utilized as carriers of new tumour therapy techniques (Israel et al., 2020). Nanoparticle therapies like these have the potential to treat multiple symptoms at once, have fewer adverse effects, and are more effective overall (Singh et al., 2015).

In addition, numerous Nano-particular medical imaging technologies have enhanced clarity and accuracy, allowing for more precise tumour identification (Singh et al., 2015; Tao et al., 2015). Thanks to developments in nanotechnology, medical nanoparticles can be created by combining various metals (such as gold, silver, iron, or liposomes) with different biological substances (Gusić et al., 2014; Tao et al., 2015). Researchers are examining all of the physical and chemical benefits that nanotechnology may bring to humanity. (Gusić et al., 2014). Because of their biological properties and the modifications made to those features, nanoparticles can be loaded with drugs and delivered to the precise target without affecting the surroundings, cryosurgery can be performed, and imaging techniques can be improved and clarified, all of which contribute to the practical, rapid, and accurate diagnosis and treatment of cancer (Adeel et al., 2020). To treat a wide range of diseases, nanomedicine is a promising new area of research. This editorial will focus on the potential applications of nanotechnology in treating various bone disorders. Bone is a tissue that aids human mobility and contributes to mineral homeostasis. However, unlike other tissues, it can regenerate during repair processes without forming scar tissue (Johnell and Kanis, 2004). Osteoporosis is a disease characterized by a loss of bone matrix that affects nine million people annually around the world. Health agencies will need to invest billions in combating the problem. Bisphosphonates (BPs) have emerged as an effective treatment in recent years (Fazil et al., 2015), however, there is still a problem with precisely delivering the medicine to the right place at the right time for the right effect. Another potentially lethal medical condition is osteomyelitis which typically occurs in patients with significant bone abnormalities (Gu et al., 2013; Fazil et al., 2015).

Bacterial *Staphylococcus aureus* infections and significant bone damage or fixation surgery cause osteomyelitis. Damaged bone’s operative excision, antibiotic therapy, and grafting are all common treatments for infected bone defects (Lu et al., 2016). Unfortunately, these drugs are not enough to keep the infection at bay (Lu et al., 2016). Therefore, there is an urgent need for a treatment that can eliminate the disease and stimulate the growth of new bone. Twenty-four million people are expected to die from cancer worldwide by 2030, making it the leading cause of death worldwide (Snoddy and Jayasuriya, 2016). . Since there is a close connection between malignant cells and bone marrow, bone metastasis is recognized as a type of bone cancer (Snoddy and Jayasuriya, 2016). Therapeutic options for bone tumors include radical resection, surgery, radiation, chemotherapy, or a combination of these modalities. However, these treatments come with a number of potentially harmful outcomes, including cancer cell resistance to anticancer medication, non-target drug-related side effects, local cancer recurrence, amputation in severe cases like osteosarcoma, and a diminished capacity for the formation of new bone (bone regeneration)) (Khan F. U. et al., 2016; Bray et al., 2018).

### Nanomedicine

Richard Feynman was the first to have a vision of the implication of nanotechnology in medicine. In 1959, he pioneered the top-down approach to nanotechnology with his lecture “There is plenty of room at the bottom.” This sparked a wave of innovation in the production and application of nanomaterials across sectors like transportation, energy, the environment, medicine, manufacturing, pollution control, *etc.*, . Multiple research types in Nanomedicine benefit humanity in diagnosing and treating tumours (oncology) and various CNS and cardiovascular infections and diseases (James et al., 2014; Zazo et al., 2016). Nanoparticles are the subject of over 80% of Nanomedicine research publications (James et al., 2014; Kannan et al., 2014). Hard and soft nanoparticles are two types of medicinal nanoparticles. Liposomes, dendrimers, micelles, and polymer particles are examples of soft nanoparticles. Inorganic and metallic structures make up complex nanoparticles (Saptarshi et al., 2013; Kannan et al., 2014). Nanomaterials can be modulated by changing their diameter, appearance, porosity, and surface load by cellular absorption and bio distribution (Gong et al., 2015).

Various nanostructures, such as gold, iron oxide, calcium phosphate, mesoporous silica, and chitosan, are under investigation for bone treatment. These could be utilized for targeted medication transport and on-spot release (Cole et al., 2011). A possible way to treat osteoporosis is to use nanomaterial to load the medicine and release it to the targeted site; BPs are more suitable for the purpose they form a connection between the nanomaterial and the bone matrix because the calcium phosphate is attracted to the bone (Holyoak et al., 2016). An alternate way is the usage of nanostructures which are charged with specific osteoclast inhibitors (bone-resorbing cells) and osteoblast promoters (bone-forming cells) to control the function of bone cells and prevent osteoporosis from occurring. Targeted medications can be delivered to the metastasized bone using BP-operated nanoparticles, increasing treatment efficacy while reducing side effects in non-target areas. Several research initiatives used poly (lactic-glycolic), hyaluronic acid, and chitosan to produce clinic nanoparticles for the specific transport of medications, hormones, or genes at the desired bone site (Farokhi et al., 2016). Combined with chondroitin sulfate and chondrocyte affinity peptide, known for cartilage targeting, these nanoparticles allow for focused delivery of inflammatory prohibitory. Using medications with osteo-inductive and antibacterial capabilities as a coding arrangement in nanoparticles is a valuable method for treating osteomyelitis. Nanoparticles could be linked with multiple medicines in several ways, including encapsulation, coating, adsorption, or binding; restoration of bone is a challenging procedure that is hampered by poor or no vessel growth and a lack of bone mineralization.

For more than a decade, researchers have been concentrating on a single growth factor, vascular endothelial growth factor (VEGF). (Sajid and Płotka-Wasylka, 2020). Nanoparticles offer promise as a valuable strategy for improving bone healing in combination or multiple drug delivery systems. On the flip side, these approaches mitigate the risks of overdosing and toxicity by minimizing the effects of ineffective dual delivery of medical goods and uncontrolled release of therapeutic agents. Therefore, it is critical to fully optimize a multi-delivery system by discovering the optimal combination of drugs or growth factors and employing the appropriate dosage to promote bone regeneration. (Sajid and Płotka-Wasylka, 2020).

### Characterization and fabrication of biomedical nanoparticles

Nanomedicine involves three distinct types of nanostructures distinguished by their diameter, appearance, and unique chemical or physical capabilities: metal nanoparticles, non-metal nanoparticles, and composite nanoparticles. For nanoparticles to serve their intended purposes in a wide range of contexts, it is essential to use the best preparation method possible (Sajid and Płotka-Wasylka, 2020). Both bottom-up and top-down processes can be used to categorize the procedures involved in preparing nanoparticles. Using this bottom-up approach, nanoparticles are produced by stacking increasingly smaller layers of essential elements. Several examples of the production of pharmaceutical nanoparticles are provided in Table 3. Among the various types of nanoparticles listed above, metal nanoparticles find the most widespread application in healthcare. Nanoparticles of metal are composed of the metal itself and a derivative of that metal. The most prevalent metal nanoparticle synthesis is the sol-gel (Sol-Gel), developed in the 1990s by Japanese scientists Sugimoto et al. It is widely used in liquid-phase mono-dispersed metal oxide particles. (Al-Tayyar et al., 2020). Using chemical and physical processes to produce a uniformly distributed soil of metal ions, followed by a redox reaction to form a gel, is the premise of this strategy. Nanoparticles of metal can nucleate, grow, and settle in a gel. Therefore, to modify the size of the resulting metal nanoparticles, the experiment’s metal colloid monodispersing must be limited to the metal ions and the oxidizing/reducing substance (Al-Tayyar et al., 2020).

**TABLE 3:** Preparation and characterization of medical.

Synthesis approach	Material	Size (nm)	Method	Features	Ref
Bottom-up approaches	TiO_2_	6–33	Sol-gel synthesis	Continuous releasing of hydroxyl radicals and superoxide ions when exposed to ultraviolet rays	Al-Tayyar et al. (2020); Sajid and Płotka-Wasylka (2020)
Fe_3_O_4_	_	10	Co-precipitation	Fe_3_O_4_ can be excited by 808 nm infrared light to realize photo-thermal conversion	Zhang et al. (2014), Sajid and Płotka-Wasylka (2020)
PEG-Fe-PDA NP	_	25–43	Microemulsions	MR Imaging enhancement with pH activation, high photo-thermal efficiency, and excellent biocompatibility	Daou et al. (2006)
Magnetite NPs	_	39	Hydrothermal approach	Small-size magnetic nanoparticles with biocompatibility and superparamagnetic	McGilvray et al. (2006)
	Au NPs	8–300	photochemical method	Enhanced medical diagnostic imaging	De Carvalho et al. (2013)
Top-down approaches	Cu-Sn oxides NPs	18–40.5	Electrical wire explosion	Ability to produce reactive oxygen species	Mascolo et al. (2013)
	Magnetite NPs	12–20	Ball milling	Small-size magnetic nanoparticles with biocompatibility	Guise (2010)

Common bottom-up strategies for metal nanoparticles include co-precipitation, hydrothermal, and photochemical approaches. In a liquid medium, the co-deposition process involves simultaneous nucleation, growth, and aggregation (Coleman, 2006) for large quantities of small insoluble particles when the solution is supersaturated. The hydrothermal method controls the morphology of the resulting nanoparticles by adjusting the vapor pressure of the solution material in a liquid environment. Furthermore, top-down approaches for preparing metal nanoparticles exist, such as electric wire explosion and ball milling. Interestingly the idea behind an electrical wire explosion is that the metal atoms are vaporized and quickly cooled into the electrolyte, resulting in oxide nanoparticles (Coleman, 2006). Controlling the electrolyte content and current strength permits fine and uniform nanoparticles to be produced. Ball milling is a method for producing Nano components with adjustable sizes on a large scale with machining instruments, such as planetary gear milling, by adjusting the equipment’s grinding time and associated process guidelines (Coleman, 2006). Aside from metallic nanoparticles, this method could synthesize other types of nanoparticles.

### Targeted delivery for preventing and treating cancer bone metastasis

One of the primary causes of cancer-related death is metastasis bone, which is most likely to be a site for spreading malignancies (Russell and Rogers, 1999). Although bone tissue is the only tissue to be subjected to the location of metastasis for prostate cancer, over-metastatic breast cancers move to the bone in 70% of situations (Wen et al., 2006). As a result, treating metastasized bone carcinoma (also known as “cancer bone metastases”) is critical for patients’ long-term survival; it has also lately emerged as a significant bone ailment. According to Coleman, bones are the most frequent site of metastasis, and the disease has the best survival rates of any major organ (Coxon et al., 2008). Moreover, the statement further highlights the need for safe, effective, and targeted non-viral medications or gene carriers in treating bone-related disorders, particularly bone cancers. BPs is a drug that gained a lot of attention for treating bone. BPs are well-known and frequently used for bone diseases due to specific bone tissue affinity. Because it provides NPs to bone tissues, this property makes BPs particularly beneficial. Strengthening bones, curing, or preventing osteoporosis, and treating Paget’s bone disease are all known effects of BP(Coxon et al., 2008). However, mounting data suggests that BPs have anti-cancer properties and can be used to treat cancer bone metastases (Coxon et al., 2008). Three generations of BPs were tested (Table 4) early clinical outcomes on preventing bone metastases in the first generation.

**TABLE 4:** Anticancer effect of BPs/nanoparticles/anticancer agent complex.

Generation	Drug	Nanoparticles bound	Anticancer agents	Effects	References
1	BP clodronate	Liposomes	Clodronate	Inhibition of cell growth Decreased metastasis	Gnant (2009)
2	Zoledronic acid	PLGA	Docetaxel	Increased cellular uptake Prolonged half-life	Tripathy et al. (2004)
3	Risedronate	PLL-CD	Cyclodextrin	Prevention of bone metastasis	Singh et al. (2018)

BP, bisphosphonate; PLGA, poly lactic-co-glycolic acid; PLL-CD, Poly-L-lysine covalent beta-Cyclodextrin.

Clodronate, or BP, induced positive outcomes in people suffering from the curse of breast cancer and was tested to see how helpful it was (Singh et al., 2018). Current research suggests this BP only applies to older women who have passed menopause (Hillen et al., 2007). Similarly, Zoledronic acid, a new generation BP, has been tested and proven to play a significant role in the prevention of metastasis A5-year research showed people with multiple myeloma who received Zoledronic acid had a greater overall survival rate than those who had conventional therapy alone (P, 0.01). Various other types of research highlighted the roles played by the second-generation BPs (Zoledronic acid) in the inhibition of angiogenesis, invasion, and tumor cell adhesion across the course of the tumor, and mounting evidence shows that such medications could prevent bone metastases from developing (Nishida et al., 2006; Hillen et al., 2007). Clinical trials on patients proved a reduced serum level of a vascular endothelial ground factor (VEGF) by Zoledronic. VEGF is a critical factor in angiogenesis, suggesting that Zoledronic acid is an angiogenesis inhibitor (Nishida et al., 2006; Hillen et al., 2007). Risedronate [RIS], a third-generation BP, is currently available and is expected as less toxic and more efficient. At the same time, a recent study suggests that adding docetaxel to the RIS had no enhanced therapeutic effect on patients with prostate cancer (Nishida et al., 2006; Abunahla et al., 2019), demonstrating that BPs were the only ones with limited therapeutic impact on patients in clinical settings. Daubing (Abunahla et al., 2019) utilized poly-l-lysine covalently grafted with beta-Cyclodextrin to supply RIS with a polycationic vector (CPL-CD).

Regardless of whether PLL-CD: RIS complexes are solvent or integrated with multilayered polyelectrolyte Nano architectures, the efficacy of RIS *in vitro* cancer cell invasion was considerably improved following complexation, according to the researchers (Abunahla et al., 2019). *In vivo*, complexes in the solution state have been shown to effectively prevent cancer-induced bone metastases in animal models (Kerbel and Kamen, 2004; Abunahla et al., 2019). Improved effectiveness of second-generation BP was observed, and NPs were considered the leading cause. Effectiveness. ZOL (zoledronate) delivers docetaxel into bones because of its high bone affinity and has shown significant bone metastasis synergy (Kerbel and Kamen, 2004).

Interestingly, PLGA NPs mixed with ZOL have a higher cellular uptake than changes in cellular uptake have demonstrated PEGylated PLGA NPs. *In vitro* studies using ZOL-hankered PLGA-PEG NPs on MCF-7 and BO2 breast cancer cell lines revealed increased cell cytotoxicity, intercellular arrest, and apoptotic job. In animal studies, ZOL-tagged NPs with technetium-99 m radiolabeling had longer circulation half-life, lower hepatic absorption, and much better tumour retention at the bone site (Kerbel and Kamen, 2004).

### Oxygen capturing approach

The recent finding indicates the primary growth may reach a maximum size of 12 mm (Yoneda, 2000; Kerbel and Kamen, 2004). Because once oxygen and nutrients are plentiful, cancer can grow beyond this extent, requiring vascularization, also known as angiogenesis (Yoneda, 2000; Ma et al., 2001). An antigenic switch is triggered when tumors switch to an angiogenic phenotype, which means a cancer phenotype change in its initial formation stage is required for growth beyond 23 mm in size (Ma et al., 2001; Desai, 2012). Moreover, the antigenic transduction pathway might get activated by the upregulation of stimulators and downregulation of inhibitors, which are involved in malignant tumor growth, progression, and metastasis (Desai, 2012). However, antiangiogenic methods may not be sufficient to eradicate malignancies independently due to the excessive compensatory processes that alter the blood vessel. (Desai, 2012; Mukherjee and Patra, 2016).

New approaches and delivery mechanisms are needed because most antiangiogenic small-molecule medicines are dangerous (Ludwig et al., 1998; Paterson et al., 2012; Mukherjee and Patra, 2016). Furthermore, defective tumor vasculature brings about the inefficient distribution of antigenic inhibitors during tumor angiogenesis, resulting in a poor pharmacokinetic profile inside the tumor stroma. Due to defective tumor vasculature, antigenic inhibitors are poorly distributed during tumor angiogenesis, leading to a subpar pharmacokinetic profile within the tumor stroma.

Nanomedicine plays a critical role in this regard (Paterson et al., 2012; Tahir et al., 2016a). Nano-theranostic-based anticancer medicines can more efficiently target tumour endothelial cells because of their tiny size and the high surface-to-volume ratio (Tahir et al., 2016a; Wang W. et al., 2017). Several researchers demonstrated unique diagnostic and therapeutic techniques based on nanotechnology for cancer treatment in this circumstance. Copper, carbon, silver, gold, silica, chitosan, and peptides conjugated with antiangiogenic characteristics are some of the novel nanomaterials mentioned by Mukherjee and Patra (Wang W. et al., 2017). However, these methods have limitations, such as requiring constant external medicine or stimulation or allowing potentially harmful metals to build up inside the body. Using their soluble byproducts, the nanostructures that Zhang created obstruct the blood vessels, thereby limiting the development of tumors due to a lack of oxygen (Wilberforce et al., 2011). ]. Deoxygenation compounds based on magnesium silicide nanoparticles (MS NPs) may find application in cancer treatment (Marelli et al., 2011; Wilberforce et al., 2011). Injectable MS NPs were synthesized by the authors of this article using self-propagating high-temperature synthesis in an O_2_/Ar mixed-gas environment. Excess O_2_ reacted with the release of heat with excess Mg, where a combination of reactions involving Si and Mg formed, resulting in Mg_2_Si/MgO composite particles possessing a core-shell shape (Mg_2_Si core within a thin outer MgO shell). Mg_2_Si was obtained by refining (Mg_2_Si/MgO by removing MgO particles (which could inhibit the undesired growth of grains) before being treated with poly vinyl-pyrrolidone (PVP) to act as a deoxygenation agent (DOA) and to prevent tumor capillaries from being re-oxygenated (Pareta et al., 2008; Marelli et al., 2011). Tumor growth was dramatically slowed in a mouse model. Researchers believe that a byproduct of nanomaterials can obstruct the veins, cutting off air to the tumor and preventing its growth.

Mg_2_Si NPs extract oxygen from the tumour cell and release SiO_2_, which helps to deal with the undesired arteries, maintain hypoxia intratumorally, and not even cause long-term toxicity (Pareta et al., 2008). As a result, developing materials with specific qualities like oxygen capture and utilizing their byproducts to block unnecessary vessels could lead to advanced antiangiogenic therapy. Novel biocompatible nanomaterials based on silicon dioxide and manganese-based materials for use as deoxygenation agents are needed due to the limitations of current treatment techniques (Pareta et al., 2008). Any way for synthesizing these materials as deoxygenation agents are worthwhile if the byproducts are biocompatible or can be safely eliminated from the body. Oxygen from the tumour microenvironment can be absorbed by the substance (composite) (1 mol of material in its composite byproduct form could have the ability to consume 2 mol of oxygen molecules or even more) (Pareta et al., 2008).

### Osteosarcoma

Even though osteosarcoma requires safe and stable techniques to replace traditional methods, including chemotherapy, surgery, and radiotherapy, nanotechnology’s implication for drug delivery is remarkable. Federman identified an antigen associated with osteosarcoma cell surface (ALCAM) and alpha-AL-HPLN, a tailored anti-ALCAM-hybrid polymerized liposomal nanoparticle immune conjugate (Butoescu et al., 2009). Scientists used this nanoparticle to transport DRX to osteosarcoma cells selectively. They found that it was more effective at killing cancer cells than either untargeted hybrid polymerized liposomal nanoparticles or conventional liposomal DXR production. Furthermore, on the application of a magnetic field to the magnetic arsenic trioxide nanoparticles, targeted effects were observed on the osteosarcoma cells (Butoescu et al., 2009; Daubiné et al., 2009), and the calcium phosphate nanoparticles were observed to possess abilities to deliver anticancer drug (cisplatin) and exhibiting dose-dependent cytotoxic effects on a murine osteosarcoma cell line (K8). Compared to DXR alone (Daubiné et al., 2009) described biocompatible nanoparticles suggested for treating osteosarcoma, the nanoparticles were lipid-modified and dextran-based. Sun et al. demonstrated that the NPs loaded with DXR had a curative effect on osteosarcoma cells which were multi-drug resistant. Still, the cells accumulated the drug in their nucleus at an increased rate and increased apoptosis in osteosarcoma cells.

Researchers used a single dextran-poly ethylamine (PEI)-NP to show that chemotherapy and gene therapy are interchangeable. DXR and PEI were grafted onto a dextran chain, and plasmid DNA could also be loaded. Since the negatively charged plasmid can be loaded with the positively charged PEI, this is a valuable process. At 8 mg/mL, the DEX-PEI dose used increased cytotoxicity in MG 63 and Saos-2 while still allowing for over 65% cell viability (Daubiné et al., 2009). Further to that, this exploration also demonstrated that NPs could efficiently transfer the plasmid pEGFP-N1 into osteosarcoma cells with minimal cytotoxicity. Therefore this device holds promise for treating patients with osteosarcoma through chemotherapy and gene therapy. (Daubiné et al., 2009).

### Osteoarthritis

**FIGURE 4:**
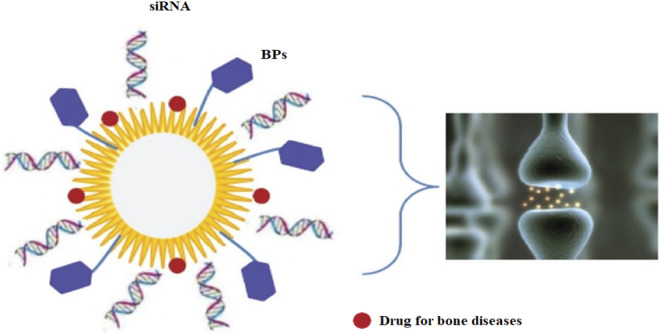
A biodegradable (polymer-based) carrier delivers NPs locally in bone. Notes: A schematic diagram shows an ideal NP capable of conveying a range of cargos, including molecules with affinity for bone tissue or cells, such as BPs, siRNA for gene therapy, and medications for bone diseases. [Reprinted with permission from International Journal of Nanomedicine at 2013].

For the treatment of osteoarthritis, nanoparticles have been under continuous study for their ability to deliver the drug to the targeted site. In this case, nanoparticles could efficiently deliver medicinal medications required to treat osteoarthritis. As well as this strategy also allows the drugs to be there in the desired cells or tissues for extended periods, as shown in Figure 4 (Gu et al., 2013). While ironically cross-linked with NP in synovial fluid, cationic polymeric hydrogels, for example, have been demonstrated to cause an impossible increase in the retention period of a model drug, “dextran."

These nanoparticles show more biodegradability than polymer-based nanoparticles or other variants of the inorganic NPs, such as stacked double hydroxides (Gu et al., 2013). Multifunctional NP carriers could be made from them. However, they will be devoid of silica to improve the roughness of the joint contact surface for local delivery. Moreover, the letters NP, siRNA, and BP stand for nanoparticle, short interfering ribonucleic acid, and bisphosphonate, respectively. As discussed earlier, NPs enhance the retention period, so they did to the IL-1 receptor antagonist (IL-1Ra) when a protein inhibitor of IL-1 was delivered locally after being conjugated onto the nanoparticles’ surface. IL-1Ra and IL-10 expressing DNA sequences successfully induced positive outcomes by reducing osteoarthritis development in a rabbit model using a CS vector (Daubiné et al., 2009). Apart from medicine administration, NPs have been studied in the imaging of arthritis; for example, in a rat arthritis model, for MRI, ultra-small superparamagnetic iron oxides were used (Daubiné et al., 2009; Salerno et al., 2010; Chaudhari et al., 2012). Another study found that gold nanoparticles might be utilized to detect IL-1 beta levels in synovial fluid. Therefore this could be applied in osteoarthritis diagnosis in conjunction with a fiber-optic particle Plasmon resonance (Pignatello et al., 2009; Salerno et al., 2010). Synthesis of a Nanofibrous scaffold was possible only using nanofibers, which helped construct customized meniscuses, eventually improving cell survival (Pignatello et al., 2009; Clementi et al., 2011).

### The advantages of local delivery in bone tissue

To treat various bone disorders, local drugs could be supplied and released to the specific area by utilizing nanotechnology. Here are the significantly advanced days of local delivery: 1) maintaining and maintaining local longer treatment times and greater efficiency (Clementi et al., 2011; Hartono et al., 2012) keeping and maintaining local longer treatment times and higher efficiency; 2) lowering side effects caused by the strategy to neighboring cells, organs, or tissues; 3) lowering the local dose in comparison to supply. Magnetic NPs are reported to be known for local distribution. Magnetic fields can detect the presence of such NPs in defined regions of bone. However, the selectivity of these nanomaterials for their intended cells is low, and a sustained magnetic field is required. (Hartono et al., 2012). Furthermore, a study was conducted by Pareta, highlighting (Schluep et al., 2006). After 5 and 8 days of culture, gamma-Fe_2_O_3_ magnetic naps were found to significantly increase the osteoblast cells’ density compared to controls (no particles). Calcium phosphate was also the constituent of magnetic nanoparticles, as it helps to cure various bone ailments. For the reduction of aggregation of these magnetic nanomaterials, coatings of citric acid and bovine serum albumin were used (Schluep et al., 2006). Therefore, the results of this coating showed that magnetic NPs, particularly (gamma-Fe_2_O_3_), increased the density of osteoblast cells in the presence of bovine serum albumin significantly increased osteoblast density after 1 day.

More study into a wide range of bone diseases is justified after this study found that gamma-Fe_2_O_3_ magnetic NPs coated with calcium phosphate increased osteoblast density compared to no particulate matter. For the *in vivo* treatment of inflammatory diseases like arthritis, researchers have described the co-encapsulation of SPIONs (Super-paramagnon-nitric iron oxide NPs) into PLGA micro-particles. To maintain the viability of the microscopic particles, SPION was tested in conjunction with an external magnetic field (Jin et al., 2010). ]. Fluorescent cell type activation and morphological characterization of micro-particle-exposed cells showed that the phagocytic process takes up these tiny particles, proving their biocompatibility. Since there were no signs of inflammation in the joints, these Nanocarriers show promise as a safe, intra-articular retainable magnetic drug delivery for treating various musculoskeletal disorders, including osteoarthritis and arthritis (Jin et al., 2010; Guo et al., 2016). The Food and Drug Administration has approved polylactic acid (PLA) and polylactic acid (PLGA) to produce nano- and micro-particles. HA is a naturally occurring polysaccharide that binds to the CD44 cell receptor in the joints (especially chondrocytes). Using PLA, PLGA, and HA, Zille disclosed a system for local drug delivery (Guo et al., 2016). In this study, arthritis was treated, and medications were carried *via* intra-articular injections of poles (D, L-lactic acid) (PLA) or LPGA coated with chemical-esterified amphiphilic HA. Targeting and biodistribution were expected to be aided by the increase in cell-interaction NPs made possible by the HA coverage. The cytotoxicity of the NPs is tested in the analyzers. Injections of dextran-FITC NPs were driven into the knees of healthy male rats once or twice weekly. There was, however, no discernible difference in the mRNA expression levels of selected early cytokines (IL-1beta and tumour necrosis factor-alpha) between control and NP-treated rats. In light of these results, they decided to test the NPs in rat models of osteoarthritis and arthritis; however, the results of these tests were not reported (Tan et al., 2010). Another scheme that has to be detailed yet is using scaffolding nanomaterials to load the medicine and gently release it locally.

### Nanotechnology in bone regeneration

Sudden traumas, fractures, infections, deformities, aging, or physical deterioration, cause specific abnormalities and malfunctioning of bone, which are severe issues for surgeons. Nanotechnology aids in treating such cases as it plays a significant role in bone regeneration. Because of research efforts targeted at optimizing the tissue–material response after implantation, success has been achieved in repairing bone and its regeneration while utilizing bioactive nanomaterials. Moreover, standard approaches for synthesizing bioactive nanostructured scaffolds that aid bone repair involve creating NPs/polymer composite scaffolds. Furthermore, the second consists of creating glass scaffolds containing small (Nano-sized) holes. Nano-sized HA was used to make NPs/polymer composite scaffolds (Pignatello et al., 2009) beta-tricalcium phosphate (Schluep et al., 2006) bioactive glasses (Tan et al., 2010), and CaSiO_3_ particles were mainly incorporated into the polymer matrix (Federman et al., 2012). These bioactive nanomaterials significantly improved the strength, degradation, ability to mineralize, and cytocompatibility of the scaffolds. Nanocomposites have a different functioning than micro composites in terms of functionality.

As a result, it is a potential choice to incorporate bioactive NPs into biopolymers to employ them in bone regeneration by enhancing their physiochemical and biological properties. Aside from the nanocomposite, the inherent nanostructure of bioactive materials is critical for enhancing bioactivity. Yan et al. (Federman et al., 2012) in 2004, combined the sol-gel technique and supramolecular surfactant techniques to synthesize a class of mesoporous bioactive glasses (MBG) to boost the bioactivity of traditional bioactive glass for bone repair by combining medication delivery with bioactive materials, their research has paved ways for utilizing Nano methods to regenerative medicine. These materials feature a highly organized mesoporous channel containing small holes of size between 5 and 20 nm and are made up of CaO-SiO_2_-P_2_O_5_. The MBG has more beneficial features and properties, such as a higher surface area, raised pore volume, and more abilities to enhance *in vitro* apatite mineralization in simulated bodily fluids, with better cytocompatibility when compared to traditional no mesoporous bioactive glasses (Barroug et al., 2004). For utilization in bone engineering and targeted drug delivery to enhance bone regeneration, nanotechnology could also create MBG as 3D porous scaffolds (Sun et al., 2011) MBG scaffolds can currently be prepared in three ways.

Moreover, the Porogen technique created the first MBG scaffold Yun et al. (Abdal-hay et al., 2013). MBG scaffolds Porous, containing various small holes with a size of 100 m, were created using methyl cellulose as the porogen. Besides, the polymer template approach, which is commonly utilized, was employed to develop the second scaffold. A wide range of MBG scaffolds with various compositions was produced to deliver the drug on the target site and tissue engineering regarding bone (Im et al., 2012).

**FIGURE 5:**
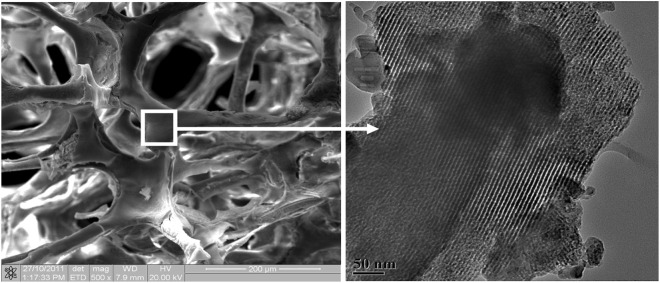
Porous mesoporous bioactive glass scaffolds with large pores (several hundred micrometers, left) and well-ordered mesoporous channel structures (5 nm, right). [Reprinted with permission from International Journal of Nanomedicine @June 2013].

Platforms with large pore sizes (300–500 m) and well-organized mesoporous containing small holes (size 5 nm) are present in the produced scaffolds. MBG scaffolds using a polyurethane sponge as a template have advantages such as high interconnectivity pore architectures and controlled pore size (porosity). In contrast, the material’s low mechanical strength is a downside. For synthesizing porous MBG scaffolds, a 3D plotting technique (direct writing or printing) is applied for enhanced properties like controlled porosity, pore size, and pore morphology. Under mild settings, the scaffold designs could be controlled by layer-by-layer plotting, which is a significant advantage of this approach for the synthesis of multifunctional MGB scaffolds, a modified 3D-printing technology employing polyvinyl alcohol as a binder was recently used; it helped control the properties like pore architecture, mechanical strength, and ability to mineralize which aids in bone regeneration application. Compared to MBG scaffolds made using conventional polyurethane foam templates, those produced using the 3D printing technique are approximately 200 times stronger. They feature a highly controlled pore design, superior apatite mineralization ability, and long-term drug delivery (Marelli et al., 2011; Yallapu et al., 2011). MBG scaffolds have the potential to deliver drugs and growth factors effectively. Dexamethasone (DEX) was loaded into MBG scaffolds, and it was discovered that prolonged DEX release dramatically increased alkaline phosphatase (ALP) activity and gene expressions in osteoblasts (ALP, bone sialoprotein, and Col I) (He et al., 2011; Marelli et al., 2011; Yallapu et al., 2011) as shown in Figure 5 (Gu et al., 2013).

**FIGURE 6:**
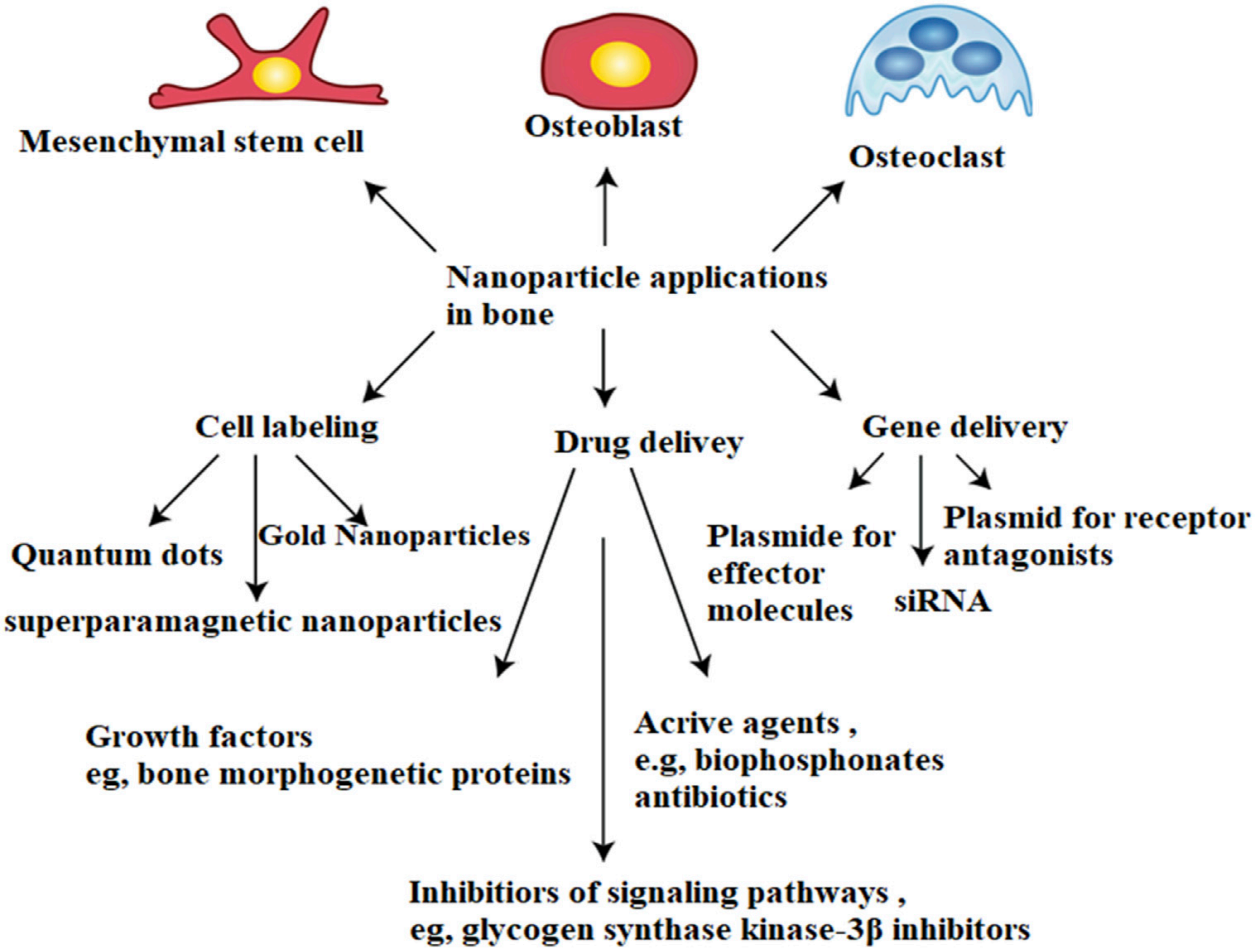
Overview of nanoparticle applications in bone.

These findings enlighten that MBG scaffolds loaded with DEX could be employed in tissue engineering as it shows great potential as a release method for enhancing osteogenesis. The effect of VEGF delivery from MBG scaffolds on endothelial cell viability was further investigated, and it was discovered that the mesoporous structures in MBG scaffolds play an important role in maintaining VEGF bioactivity, further improving endothelial cell viability, indicating that MBG scaffolds are an excellent VEGF carrier for stimulating angiogenesis (Jo et al., 2009). As a result, MBG scaffolds, as a typical Nano biomaterial, harness their unique Nano pore structure to combine drug delivery and bioactivity to enhance their applicability in the regeneration of bone, a new approach in the regard of tissue engineering is the bioactivity and functionality of nanomaterials in targeted drug delivery. The strength, ability to mineralize degradability, and cytocompatibility of polymer skulls were increased by these bioactive nanomaterials. Nanocomposites serve a different purpose than micro composites (Jo et al., 2009; Zhang et al., 2012). So, to enhance the properties of both nanomaterials and biopolymers for bone regeneration, combining their physiological and biological properties is viable. Figure 6 shows an overview of nanoparticle applications in bone.

Surface targeting for bone resorption 50%–70% of the bone composition is inorganic minerals, and 20%–40% is the organic matrix. Water constitutes 5%–10%, and fats are less than 3%. Bone-seeking is best directed toward hydroxyapatite (HA), the most common inorganic component of bone tissues (Luhmann et al., 2012; Zhang et al., 2012). At the bone resorption surface, where the osteoclasts are found, the HA is highly crystalline. (Luhmann et al., 2012). Anionic ligands are typically chelated with superficial calcium ions on the resorption surface to target bone (Cole et al., 2016). Phosphate- and carboxylate-rich compounds are the ligands that are considered to be designed for bone targeting. Few small drugs (Hochdorffer et al., 2012; Cole et al., 2016; Tauro et al., 2017; David et al., 2019), macrocyclic chelators (Minoshima et al., 2019; Zaman et al., 2023), fluorescent dyes (Liu et al., 2008; Bao et al., 2015; Zaman et al., 2023), nucleic acids (Liu et al., 2008), cyclodextrin (Liu et al., 2008), fullerene C_60_ (Kubícek et al., 2005)[230], proteins (Kubícek et al., 2005), polymers, nanomaterials, and cells were conjugated to the ligands to endow these molecules and species with bone-homing properties (Gonzalez et al., 2002).

Bisphosphonates and analogs: BPs 1) are the most widely used bone-targeting ligands (Miller et al., 2009). Moreover, the purpose of binding calcium ions to HA Bidentate requires two phosphonate groups. On BPs, the R_1_ group significantly impacts bone binding affinity. In most BPs, R_1_ is a hydroxyl group. BPs experience more binding relationships owing to the tridentate binding of the hydroxyl group. To prevent the formation of osteoclast cells and metastases, BPs have been approved for clinical use 2), clodronate 3) and tiludronate 4) are non-nitrogen-containing BPs; alendronate (ALN, 5), Pamidronate 6), and ibandronate 7) are nitrogen-containing BPs; and zoledronate (ZOL, 8) and risedronate 9) are nitrogen-heterocycle-containing BPs. The hydroxyl groups on HA (Miller et al., 2009; Riehemann et al., 2009) could create hydrogen bonds with the nitrogen group at R2. Even though hyperthermic procedures have been explored for the treatment of bone metastases for a long time, doctors have been cautious about utilizing them for various reasons: Because of the thickness of the cortical bone, they have poor thermal conductivity, and the medulla is heavily vascularized. Until recently, radiotherapy was employed to treat bone metastases.

Although radiotherapy for treating bone metastases is generally safe and efficient, it can occasionally cause soft tissue harm, and cancer cells are not permanently destroyed (Korkusuz, 2013). In innovative hyperthermic procedures, magnetic materials in an electromagnetic field are increasingly utilized. Microwaves (Matsumine et al., 2011), lasers (Vogl et al., 2001), and radio-frequency ablation (Groenemeyer et al., 2002) have all been shown to produce thermotherapy. Since 2003, this treatment has been examined in clinical studies (Matsumine et al., 2007). A recent study examined how heat treated 23 people with bone metastases. In 32 percent of patients, lesions were decreased, and new bone formation was generated; however, in 64 percent of lesions, there was no additional growth for 3 months, and the surgery failed in only one case (4%). These results were statistically better than those acquired only through surgical techniques. On the other hand, patients who received surgery and postoperative radiation had similar outcomes. This demonstrates that surgery is as effective as a novel hyperthermic treatment paired with radiation.

#### Redox responsive nanocarriers

Utilization of stimulus responsiveness is a new approach in the treatment of OSA. These stimuli include redox potential, tumour acidity, and enzymes that cause drug release on the tumour site (Kanamala et al., 2016). For OSA-focused delivery, redox-sensitive NPs are currently being researched. Based on this notion for medicine administration in the OSA, liposomes with hyaluronic acid were developed, which were redox-sensitive and used the tumour’s redox potential as stimuli. These were focused on CD-44 receptors to improve OSA chemotherapy. After that, PEG and cholesterol were used to stabilize the liposomes. Doxorubicin was used as a model drug for cytoplasmic drug transport in the instance of OSA. PEG-cholesterol coupled hyaluronic acid (HA) liposomes suppressed tumour growth while lowering liver uptake compared to ordinary liposomes. As a result, the CD-44 targeted intracellular drug delivery vehicle has demonstrated its potential (Chi et al., 2017). Feng et al. also discovered that an OSA-targeting liposome system functionalized with a redox-cleavable dual-targeting polymer, bone, and cluster of differentiation 44 is effective (CD44). Feng and others demonstrated the internalization of RGD by a tumor-penetrating peptide. In this context, Alendronate (ALN), a chemical for bone targeting, was initially conjugated with HA, a CD44 ligand. This ALN-HA conjugation was combined with DSPE-PEG2000-COOH *via* a bio-reducible disulfide linker (-SS-) to form ALN-HA-SS-L, a functionalized lipid that could be injected into DOX-loaded liposomes after they were prepared. In addition to solid and quick cellular absorption, ALN HA-SS-L-L/DOX demonstrated significantly more potent cytotoxicity for human OSA MG-63 cells than different reference liposomes. ALN-HA-SS-L-L/DOX exhibited a significant reduction in tumour growth and increased survival time in orthotropic OSA nude mice models.

This shows that ALN-HASS- L-L/DOX, a dual-targeting therapy for bone and CD44 with redox sensitivity, could be an effective OSA-targeted treatment. Internalizing RGD can be used in conjunction with other medications. Another study developed a cationic liposomal estrogen-related method for targeted delivery to OSA. Choto oligosaccharides were covalently attached to liposomes *via* disulfate linkage for estrogen receptor targeting, whereas estrogen was anchored *via* PEG. In addition, the liposomes included doxorubicin, which was used to treat OSA. The liposomes released the medication in response to tumoral intracellular glutathione. Tumour targeting was investigated using the uptake of MG-63 OSA cells. Choto oligosaccharides also grafted estrogen-functionalized cationic liposomes into OSA cells, delivering intracellular medicine to the estrogen receptor (Chi et al., 2017; Yin et al., 2018). Other Choto oligosaccharides surface-coated redox-sensitive fusogenic liposomes were used to explore OSA. Doxorubicin, an antitumor medication, was added to the liposomes. These liposomes were shown to be more stable, with less drug leakage and more cytotoxicity in cancer cells.

The redox-sensitive liposomes were more hazardous to MG-63 OSA cells than standard liposomes but less toxic to LO_2_ liver cells. Liposomes improved animal survival rates in general (Yin et al., 2017; Yin et al., 2018). Another study used CD133 aptamers on the surface of hybrid NPs made of lipid and polymer mixtures to deliver all-trans-retinoic acid to OSA cells. As a result, all-tans retinoic acid was encapsulated in mixed lipid–polymer NPs to target and treat cancer precursor cells effectively. Both the tumour sphere generation and cytotoxic assays for hybrid NPs were promising. Over 144 h, all-trans retinoic acid was revealed, and the mixed NPs were internalized in OSA cells, demonstrating a novel strategy for treating OSA. Another fascinating study investigated the utilization of combined metallic and polymeric NPs to treat OSA. Copper-loaded chitosan nanoparticles with a spherical form were created. Internalization of hybrid nanoparticles was greater than that of free CuSO4. NPs also showed increased levels of mitochondrial ROS and caspase-3 activity (Ai et al., 2017).

## Conclusion

Since the advent of nanotechnology in materials science, NP-based drug delivery systems have been at the forefront of cancer research. To maximize their effectiveness against cancer, nano-therapeutics must be synthesized under tightly regulated conditions, and their physiochemical behavior and surface activity must be highly tuned. Cancer therapy relies heavily on NPs targeting of cancer cells and nano-bio interfacial exchanges with cancer cells. Because of the wide variety of biological barriers that cancer patients face, this review needs to delve into synthesizing various NPs optimized for cancer treatment and drug delivery. Suitable barriers in drug delivery, as well as the possibility of combining hydrophilic and hydrophobic substances and admiration *via* various oral and inhalation routes, are being taken into account by the NPs. Surface area, size, charge, and shape all play essential roles in the delivery of drugs. However, only a tiny number of Nano drugs are currently available for use in cancer therapy due to concerns about their toxicity and *in vivo* performance. Nanomedicine has a long way to go before it can provide innovations that can significantly improve cancer treatment, but further development in this field will bring about such innovations. At this point, it is possible to foresee that the next-generation of Nanomedicine will improve nanomaterials by employing innovative nanoparticle designs and strategies that can lead to more effective cancer treatment plans.
